# The Impact of COVID-19 Pandemic and Lockdown Restrictions on Cardiac Implantable Device Recipients with Remote Monitoring

**DOI:** 10.3390/jcm10235626

**Published:** 2021-11-29

**Authors:** Igor Diemberger, Alessandro Vicentini, Giuseppe Cattafi, Matteo Ziacchi, Saverio Iacopino, Giovanni Morani, Ennio Pisanò, Giulio Molon, Tiziana Giovannini, Antonio Dello Russo, Giuseppe Boriani, Emanuele Bertaglia, Mauro Biffi, Maria Grazia Bongiorni, Roberto Rordorf, Giulio Zucchelli

**Affiliations:** 1 Department of Experimental, Diagnostic and Specialty Medicine, Alma Mater Studiorum—University of Bologna, 40126 Bologna, Italy; 2 UOC di Cardiologia, IRCCS Policlinico S.Orsola-Malpighi, 40138 Bologna, Italy; matteo.ziacchi@gmail.com (M.Z.); mauro.biffi@unibo.it (M.B.); 3 Cardiac Intensive Care Unit, Arrhythmia and Electrophysiology and Experimental Cardiology, Fondazione IRCCS Policlinico San Matteo, 27100 Pavia, Italy; a.vicentini@smatteo.pv.it (A.V.); r.rordorf@smatteo.pv.it (R.R.); 4 Cardiologia 3, Dipartimento Cardiotoracovascolare, ASST Grande Ospedale Metropolitano Niguarda, 20162 Milano, Italy; giuseppe.cattafi@ospedaleniguarda.it; 5 Maria Cecilia Hospital, 48033 Cotignola, Italy; iacopino@iol.it; 6 UOC di Cardiologia, Azienda Ospedaliero Universitaria Integrata di Verona, 37126 Verona, Italy; Giovanni.morani@aulss7.veneto.it; 7 UOC di Cardiologia, Ospedale Vito Fazzi Lecce, 73100 Lecce, Italy; cardiologia.polecce@ausl.le.it; 8 UOC di Cardiologia, IRCCS Ospedale Sacro Cuore Don Calabria, 37024 Negrar, Italy; giulio.molon@sacrocuore.it; 9 UOC di Cardiologia, Ospedale Misericordia e Dolce, 59100 Prato, Italy; tgiovannini@usl4.toscana.it; 10 Cardiology and Arrhythmology Clinic, University Hospital “Ospedali Riuniti”, 60126 Ancona, Italy; antonio.dellorusso@ospedaliriuniti.marche.it; 11 Cardiology Division, Department of Biomedical, Metabolic and Neural Sciences, University of Modena and Reggio Emilia, Policlinico di Modena, 41125 Modena, Italy; giuseppe.boriani@unimore.it; 12 Department of Cardio-Thoraco-Vascular Sciences and Public Health, Azienda Ospedaliera Universitaria di Padova, 35128 Padova, Italy; bertagliaferro@alice.it; 13 Second Division of Cardiology, Cardio Thoracic and Vascular Department, Azienda Ospedaliero Universitaria Pisana, 56126 Pisa, Italy; m.g.bongiorni@med.unipi.it (M.G.B.); g.zucchelli@ao-pisa.toscana.it (G.Z.)

**Keywords:** COVID-19, pandemia, physical activity, arrhythmias, heart failure, telemonitoring

## Abstract

From 2020, many countries have adopted several restrictions to limit the COVID-19 pandemic. The forced containment impacted on healthcare organizations and the everyday life of patients with heart disease. We prospectively analyzed data recorded from implantable defibrillators and/or cardiac resynchronization devices of Italian patients during the lockdown (LDP), post-lockdown period (PLDP) and a control period (CP) of the previous year. We analyzed device data of the period 9 March 2019–31 May 2020 of remotely monitored patients from 34 Italian centers. Patients were also categorized according to areas with high/low infection prevalence. Among 696 patients, we observed a significant drop in median activity in LDP as compared to CP that significantly increased in the PLDP, but well below CP (all *p* < 0.0001). The median day heart rate and heart rate variability showed a similar trend. This behavior was associated during LDP with a significant increase in the burden of atrial arrhythmias (*p* = 0.0150 versus CP) and of ventricular arrhythmias [6.6 vs. 1.5 per 100 patient-weeks in CP; *p* = 0.0026]; the latter decreased in PLDP [0.3 per 100 patient-weeks; *p* = 0.0035 vs. LDP]. No modifications were recorded in thoracic fluid levels. The high/low prevalence of COVID-19 infection had no significant impact. We found an increase in the arrhythmic burden in LDP coupled with a decrease in physical activity and heart rate variability, without significant modifications of transthoracic impedance, independent from COVID-19 infection prevalence. These findings suggest a negative impact of the COVID-19 pandemic, probably related to lockdown restrictions.

## 1. Introduction

On 11 March 2020, the World Health Organization (WHO) declared the SARS-CoV-2 infection a world pandemic. All involved countries reacted by establishing several restrictions aimed at containing the spread of the infection. In Italy, the spread of infection was anticipated by many other countries, and the Italian government established a lockdown period from 9 March 2020 until 3 May 2020. During this period, all people were requested to stay at home, unless required to move for work, health, or other major reasons, such as assisting sick or disabled relatives or to purchase basic necessities. The forced containment obviously impacted on the amount of physical activity while increasing the psychological distress due to confinement with a plausible negative impact on patients with structural heart disease [1,2,3], especially in view of the impact of COVID-19 on healthcare resources dedicated to chronic conditions, such as heart failure. Finally, there has been a reported increase in the incidence of both supraventricular and ventricular arrhythmias in patients with COVID-19 infection [4]. Current implantable cardiac defibrillator (ICD) and devices for cardiac resynchronization therapy (CRT-D/P) are generally equipped with home-monitoring facilities, offering the unique opportunity to monitor physical activity daily, as well as heart failure diagnostic indexes and arrhythmic burdens. The aim of the present analysis was therefore to evaluate the impact of lockdown restrictions on physical activity, heart failure diagnostics, and cardiac arrhythmias in a large population of patients with heart disease monitored daily by means of ICD and CRT devices. 

## 2. Methods

### 2.1. Population

We performed a retrospective analysis on prospectively collected data from a population of ICD or CRT carriers who were followed by 34 cardiological centres in the Italian One Hospital Clinical Service Project, a national cardiovascular data repository and medical care project aiming at describing and improving the use of Medtronic cardiac implantable electronic devices (CIED) in Italian clinical practice. Inclusion criteria for this analysis were: (1) ICD or CRT implantation before November 2018; and (2) device diagnostic data being available from 9 March 2019 to 31 May 2020. Patients were also categorized into subgroups based on geographical region. We defined those at increased incidence of COVID-19 during the lockdown period as high-risk regions (Emilia-Romagna, Lombardia, Marche and Veneto), and all other regions as low-risk regions (considering an incidence >5 cases over 1000 inhabitants during the study period) [5]. The project was approved by each site’s medical ethics committee or medical director, and it conforms to the principles outlined in the Declaration of Helsinki. Each patient provided informed consent for data collection and analyses.

### 2.2. Data Collection

At the baseline visit, clinical patient characteristics were collected, including: age, sex, history of Heart Failure (HF), history of atrial tachycardia/fibrillation (AT/AF), underlying cardiac disease, NYHA class, cardiovascular risk factors, previous history of cardiovascular and thromboembolic events, thromboembolic risk index (CHA2DS2-VASc), and echocardiographic parameters. Device data were transmitted through a remote monitoring system (Carelink system, Medtronic, Minneapolis, MI, USA) every 3–6 months and automatically saved in the dedicated database. 

Collected device data included the following clinical diagnostic parameters:

- Patient activity, that is, the total time active per day using a capacitive accelerometer; a minute is considered active if the counts exceed a threshold equal to walking approximately 70 steps/min.
- AT/AF burden, measured as the total duration of a fast atrial rate during a 24 h period, with an atrio-ventricular conduction ratio ≥2:1.
- Optivol Fluid index, that corresponds to changes in thoracic fluid levels. The fluid index trend is the cumulative difference between the daily average and patient-specific reference intrathoracic impedances. The intrathoracic impedance is calculated from the voltage measured from an asynchronous current applied between the right ventricular lead and the device case. The Optivol index was represented as the number of days per week with Optivol index >60 Ohm or >120 Ohm.
- Heart rate variability (HRV), that is, the standard deviation of 5 min median of atrial intervals during a 24 h period. Reduced HRV suggests an increase in sympathetic tone.
- Night heart rate (NHR), that is, the average heart rate between midnight and 4:00 a.m. NHR is a proxy for the resting heart rate.
- Percentage of ventricular pacing.
- Number of VT episodes and number of total ventricular arrhythmic episodes, that includes monitored VT, VT, and fast VT and VF. Ventricular arrhythmias were analyzed as a mean weekly number of episode/100 patients and as a percentage of patients with at least one episode of ventricular arrhythmias.
- AT/AF was summarized by the median and total duration per day and was collected only in patients with a functional atrial lead.

### 2.3. Timeframe of the Analysis

The aim of this analysis was to describe the trends of device diagnostic data in CIED carriers during the lockdown period due to COVID-19 and to compare them with those in the same period of 2019. For these purposes, the assessment was performed considering the following four periods:

(1) Period 1 (lockdown control period): 11 March 2019—5 May 2019;
(2) Period 2 (post-lockdown control period): 6 May 2019—2 June 2019;
(3) Period 3 (lockdown period): 9 March 2020—3 May 2020;
(4) Period 4 (post-lockdown period): 4 May—31 May 2020.

### 2.4. Statistical Analysis

Descriptive statistics were used to summarize all results. These include mean and standard deviation for continuous variables, and counts and percentages for categorical variables. All plots show the mean value of each device’s diagnostic data across patients by week. On the x-axis, the start date for the corresponding week is displayed. For each continuous device’s data, linear mixed models were used to estimate the average levels in the periods of interest, along with their 95% confidence interval (95% CI). Optivol and arrhythmic episode endpoints (VT and all ventricular arrhythmias episodes) were analyzed using generalized estimating equation (GEE) models. Estimates at the average point for each period were expressed as the number of episodes/100 pts per week, along with their 95% CIs. For both types of endpoints, models include the period as a fixed factor, and the week as a repeated factor with the patient ID as subjects, and a first-order auto-regressive covariance structure. We explored differences among patients with/without arrhythmias during the lockdown period using Student’s t test or Wilcoxon’s rank sum test, as appropriate, for continuous variables; for categorical variables, the chi-squared test or Fisher’s exact test was used. The between-group difference in the number of days with Optivol above the threshold was tested using the Poisson regression model. All tests were two-sided and a value of *p* < 0.05 was considered statistically significant. SAS software, version 9.4, (SAS Institute Inc., Cary, NC, USA) was used to perform all statistical analyses.

## 3. Results

The population set satisfying the selection criteria included 696 patients, of which 501 were in high-risk regions and 195 in low-risk regions. Table 1 shows the baseline characteristics for the analyzed population. 

### 3.1. Patients’ Activity

**Figure 1:**
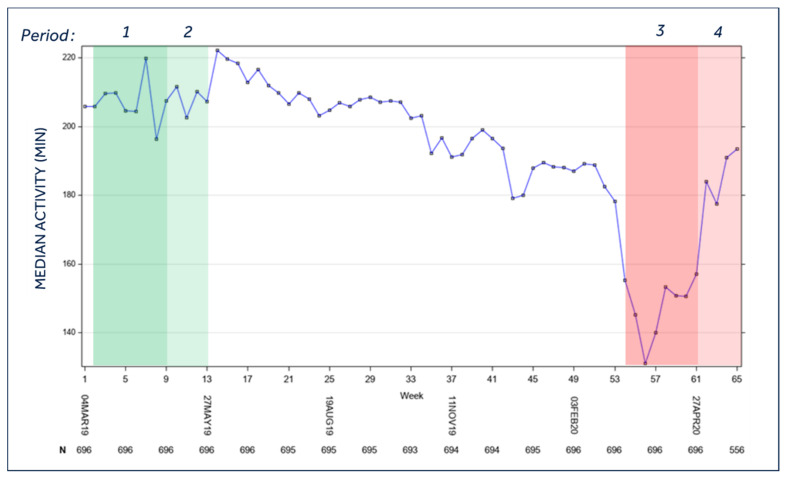
Median activity of the entire population during the study period. Legend: *Period 1* = Lockdown control period (year 2019); *Period 2* = Post-lockdown control period (year 2019); *Period 3* = Lockdown period (year 2020); *Period 4* = Post-lockdown period (year 2020). For the temporal definition of the three periods, please see the Methods section; *p* < 0.0001 for mean *period 3* vs. mean *period 1*, mean *period 3* vs. mean *period 4*, mean *period 2* vs. mean *period 4*; *p* = 0.0162 mean *period 1* vs. mean *period 2*.

As expected, the daily median activity time (Figure 1) was significantly lower in the lockdown period as compared to the same period of the previous year [period 3: 159.2 min (95%C.I. 151.1–167.2) vs. period 1: 206.9 min (95%C.I. 198.7–215.0); *p* < 0.0001]. After the interruption in daily life from the lock-down, daily activity significantly increased, but was well below the 2019 control period [period 4, 185.5 min (95%C.I. 177.3–193.8) vs. period 2, 210.9 min (95%C.I. 202.9–219.0), *p* < 0.0001]. Notably, no difference was present in the activity temporal trend between patients from high- and low-risk regions.

### 3.2. Burden of AF

**Figure 2:**
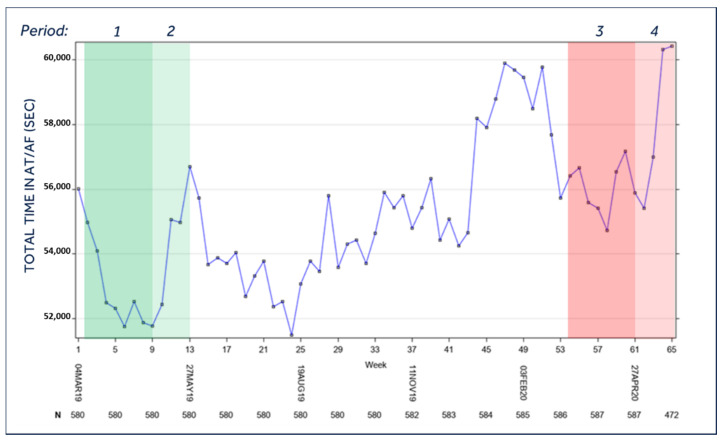
Total time in AT/AF of the entire population during the study period. Legend: *Period 1* = Lockdown control period (year 2019); *Period 2* = Post-lockdown control period (year 2019); *Period 3* = Lockdown period (year 2020); *Period 4* = Post-lockdown period (year 2020). For the temporal definition of the three periods please see the Methods section; AT/AF = atrial tachycardia/atrial fibrillation; *p* = 0.0150 for mean *period 3* vs. mean *period 1*; *p* = N.S. for all other comparisons.

Figure 2 shows the trend of total time in AT/AF for the overall population during the observation period, evidencing a significant increase in the median time in AT/AF during the lockdown period with respect to the burden recorded in the control period of the previous year (*p* = 0.0150). When AT/AF data were analyzed separately in high-risk and low-risk regions, a similar increasing trend was found in both subgroups; nevertheless, the difference between periods 3 and 1 was statistically significant only in low-risk regions (*p* = 0.0025).

### 3.3. Day and Night Heart Rate

Unsurprisingly, the median day heart rate presented a decrease during the lockdown period similarly to daily activity. Indeed, when comparing period 3 with period 1, a statistically significant (*p* < 0.001) reduction in the median day heat rates was found: 70.7 bpm (95%C.I. 70.1–71.3) vs. 72.1 bpm (95%C.I. 71.5–72.7). Notably, the variation in median day heart rate was not associated with modifications of median rate during night-time [period 3: 64.5 bpm (95%C.I. 64.0, 65.0) vs. period 1: 64.3 bpm (95%C.I. 63.8, 64.8); *p* = 0.265]. 

### 3.4. Percentage of Ventricular Pacing

Both ventricular pacing in single-chamber/dual-chamber devices [4.5% (95%C.I. 2.3%, 6.7%) in period 3 vs. 4.9% (95%C.I. 2.7%, 7.1%) in period 1; *p* = 0.0523] and in biventricular devices [94.0% (95%C.I. 92.8%, 95.3%) in period 3 vs. 93.9% (95%C.I. 92.6%, 95.1%) in period 1; *p* = 0.5246] did not change significantly between the lockdown period and the same period of the previous year neither when comparing the four periods of observation. 

### 3.5. Optivol Fluid Index

Despite the significant changes in the management of heart failure patients during the lockdown period, the Optivol fluid index graph did not show any significant difference among the four considered periods, in terms of the percentage of patients with Optivol exceeding the settled threshold (10% and 5% in the four periods, for thresholds of 60 and 120 ohm) or in terms of the mean number of days above the threshold when considering the standard nominal value of 60 ohm [58.7 (95%C.I. 49.1–70.2), 55.8 (95%C.I. 46.0–67.7), 58.8 (95%C.I. 49.2–79.2) and 53.0 (95%C.I 43.7–64.3) days/100 patients per week, respectively in period 1, period 2, period 3, and period 4; all *p* = N.S.] or a more conservative threshold of 120 ohms [23.4 (95%C.I. 17.3–31.6), 23.5 (95%C.I. 16.9–32.6), 26.6 (95%C.I. 19.9–35.5), and 22.3 (95%C.I. 16.4–30.4) days/100 patients per week, respectively in period 1, period 2, period 3, and period 4; all *p* = N.S.].

### 3.6. Heart Rate Variability

**Figure 3:**
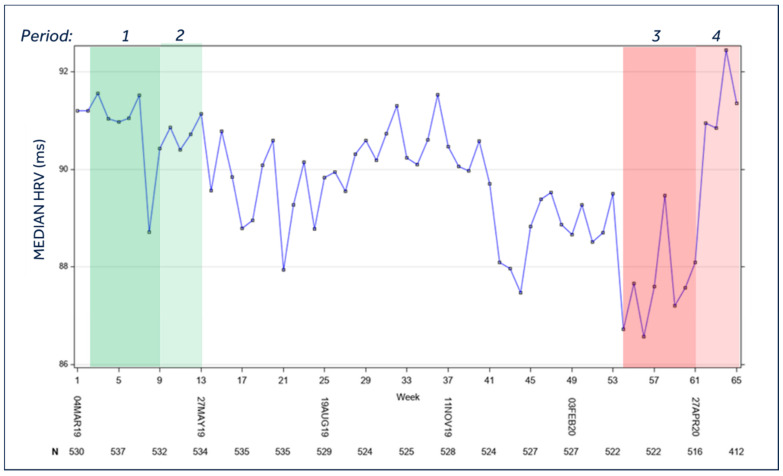
Median heart rate variability of the entire population during the study period. Legend: *Period 1* = Lockdown control period (year 2019); *Period 2* = Post-lockdown control period (year 2019); *Period 3* = Lockdown period (year 2020); *Period 4* = Post-lockdown period (year 2020). For the temporal definition of the three periods please see the Methods section; HRV = heart rate variability; *p* < 0.0001 for mean *period 3* vs. mean *period 1*, mean *period 3* vs. mean *period 4*; *p* = 0.0018 for mean *period 1* vs. mean *period 2*; *p* = N.S. for all other comparisons.

The trend of heart rate variability changed according to the modifications of physical activity and median day heart rate. We found a reduction (Figure 3) during the lockdown (period 3) with an increase in the post-lockdown period (period 4; *p* < 0.0001 vs. period 3). The comparison between period 3 (86.1 ms; 95%C.I. 83.6–88.5) and period 1 (90.4 ms; 95%C.I. 88.0–92.9) once again reached statistical significance (*p* < 0.0001).

### 3.7. Ventricular Arrhythmias

**Figure 4:**
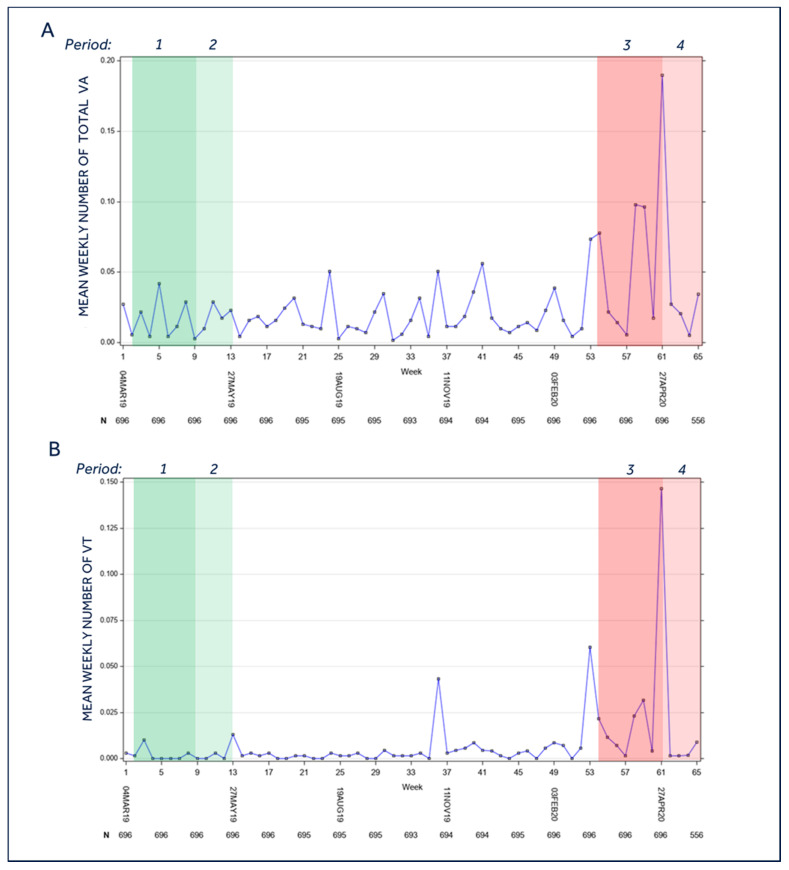
Burden of ventricular arrhythmias of the entire population during the study period. (**A**). Mean weekly number of ventricular arrhythmias (VT monitor, VT, FVT and VF); (**B**) Mean weekly number of ventricular tachycardias (VT). end: *Period 1* = Lockdown control period (year 2019); *Period 2* = Post-lockdown control period (year 2019); *Period 3* = Lockdown period (year 2020); *Period 4* = Post-lockdown period (year 2020). For the temporal definition of the three periods please see the Methods section; VA = ventricular arrhythmias; VT = ventricular tachycardias; (**A**): *p* = 0.026 for mean *period 3* vs. mean *period 1*; *p* = 0.0338 for mean *period 3* vs. mean *period 4*; *p* = N.S. for all other comparisons. (**B**): *p* = 0.024 for mean *period 3* vs. mean *period 1*; *p* = 0.0035 for mean *period 3* vs. mean *period 4*; *p* = N.S. for all other comparisons.

During the lockdown period, the overall burden of ventricular arrhythmias (VT monitor, VT, FVT, and VF, Figure 4A), showed a significant increase [period 3: 6.6 events/100 patients per week (95%C.I. 3.2–14.0) vs. period 1:1.5 events/100 patients per week (95%C.I. 0.8–2.9); *p* = 0.0026]. Restricting the comparison to VT episodes (Figure 4B), period 3 showed a significantly higher burden vs. period 1 [3.2 events/100 patients per week (95%C.I. 1.2–8.1) vs. 0.2 events/ 100 patients per week (95%C.I. 0.0–0.9); *p* = 0.0024]. No difference in VF incidence was observed amongst the four periods. These results were confirmed when the data were analyzed separately in high- and low-risk regions. Indeed, in high-risk regions, the overall burden of ventricular arrhythmias was 7.7 events/100 patients per week (95%C.I. 3.3–18.1) in period 3, vs. 2.0 events/100 patients per week (95%C.I. 1.0–4.0) in period 1 (*p* = 0.0118). Similarly, in low-risk regions, the number of VT episodes was 3.8 events/100 patients per week (95%C.I. 1.1–13.1) in period 3 vs. 0.3 events/100 patients per week (95%C.I. 0.0, 2.2) in period 1 (*p* = 0.0326). Notably, when considering ventricular tachycardias [period 3: 6.6 events/100 patients per week (95%C.I. 3.2–14.0) vs. period 4: 2.0 events/100 patients per week (95%C.I. 0.8–5.1); *p* = 0.0338] and total ventricular arrhythmias [3.2 events/100 patients per week (95%C.I. 1.2–8.1) vs. 0.3 events/100 patients per week (95%C.I. 0.1–1.0); *p* = 0.0035] we found a significant reduction in the post-lockdown period vs. the lockdown period (Figure 4).

### 3.8. Patients with/without Arrhythmias during the Lockdown

During the lockdown period, 101 patients (14.5% overall) experienced any kind of arrhythmic event (defined as AT/AF episodes with >1 h burden or any VT/VF). The clinical profile of patients with arrhythmic events during the lockdown period was similar to the remaining population, with the exception of a more advanced age and a history of any previous arrhythmic event. More interestingly, among the parameters collected by the implanted device, we found a significant decrease in physical activity (both in terms of median and total time) coupled with a tendency towards increased fluid accumulation, in terms of the number of days with an impedance above 120 ohm (Table 2).

## 4. Discussion

The present analysis evidences the effect of the COVID-19 pandemic on several parameters recorded by CIED in a multicenter cohort of unselected patients by comparing data recorded during the first wave of COVID-19 in Italy (before, during, and after the lockdown) with the same parameters collected in the previous year.

According to our findings, the patients experienced a significant increase in burdens of AT/AF and ventricular arrhythmias, associated with a decrease in physical activity and heart rate variability, without any significant modification regarding the average heart rate (only a minor albeit significant increase in daily heart rate) and amount of ventricular pacing.

Notably, after the lowering of containment measures, we also found a reduction in the burden of ventricular arrhythmias that followed the improvement in physical activity and heart rate variability. 

These findings are relevant because they highlight the effect of the societal adaptation to such a catastrophic event, including the consequences of national measure to contrast the pandemic. Previous studies evidenced the negative effects of natural disasters and psychological stress on arrhythmic burdens [6,7], but this is the first pandemic of such magnitude since the Spanish influenza pandemic of 1918 with such a widespread effect over a worldwide population. Several authors investigated the effects of the COVID-19 pandemic on comorbidities, proving that patients with cardiovascular conditions are at higher risk both in terms of contagion and worse outcomes [8]. However, this interaction is not limited to subjects with coexistence of both disorders since, after the declaration of the pandemic by the WHO on March 11 [9], several actions taken by the countries have greatly affected the overall management of patients, economics, and the lifestyle of the global population [10,11]. It has been estimated that hospital admission for acute heart failure almost halved after the COVID-19 breakout in the UK [12], usually with a more severe status, while there has been a reduction of more than one-third of admissions for acute coronary syndromes leading to a steep increase in complications (e.g., sudden death, ventricular arrhythmias, and mechanic complications) [13,14]. However, these findings can have different explanations, and the more plausible mechanism is a widespread fear of the contagion both from the population and healthcare authorities. In our population, we found a similar effect in residents in areas with a low prevalence and high prevalence of COVID infection confirming this explanation. Accordingly, these considerations prompted the development of ad hoc recommendations from principal national and international associations to manage urgent cardiovascular interventions in these settings both in COVID and non-COVID patients [15,16,17,18], while non-urgent procedures have been deferred/cancelled. However, a more interesting approach was the increase in telemedicine, especially in the field of monitoring of CIED carriers, an area that was almost up to date with such an approach [19,20]. This reason can explain why the enrolled patients seem not to have experienced any worsening in the thoracic fluid accumulation index during the lockdown period with respect to the control analyzed periods. However, a similar stability was not found in the arrhythmic profile of the patients who experienced an increase in the burden of atrial fibrillation and ventricular arrhythmias, independently from the prevalence of COVID in the leaving area. Considering the global trend in physical activity and heart rate variability, this phenomenon seems to be associated with a direct negative impact of the pandemic and of the associated limitations connected with lockdown measures. With regard to the explorative comparison between patients with vs. without arrhythmic episodes during the lockdown period, while confirming the importance of physical activity, it suggests that older subjects with a previous history of AT/AF and/or ventricular arrhythmias are at increased risk of recurrences under restrictive measures. We also found a trend in fluid accumulation which was probably less affected by restrictive measures in view of the availability of remote follow-up.

**Figure 5:**
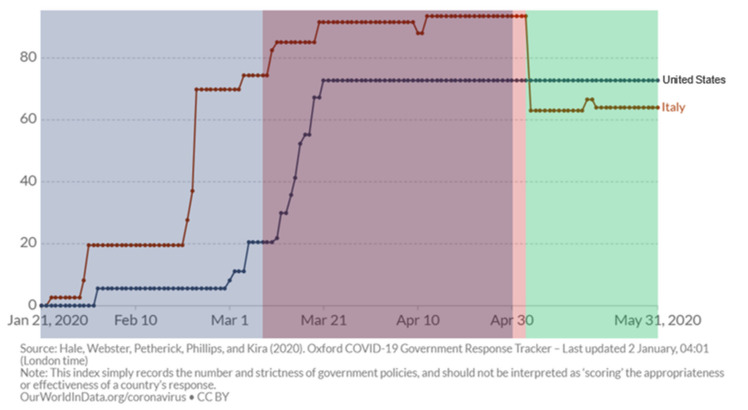
Evaluation of restricting policies in the U.S. and Italy during the observation periods analyzed by O’Shea et al. (blue shaded) and in our analysis (red-shaded area is the lockdown period, and the green-shaded area is the post-lockdown period). The y-axis reports a composite measure based on nine response indicators including school closures, workplace closures, and travel bans, rescaled to a value from 0 to 100 (100 = strictest) (for additional information, see https://ourworldindata.org/grapher/covid-stringency-index?tab=chart, accessed on 2 January 2021).

In a recently published paper, O’Shea et al. showed the results of an analysis of the burden of ventricular arrhythmias among 5963 ICD carriers enrolled by 20 centers in 13 different U.S. states, comparing a 100-day period during the COVID-19 pandemic vs. two control periods in late 2019 and early 2019 (seasonal control) [21]. They showed a progressive decrease in the burden of ventricular arrhythmias, about one-third less than the two control periods. These results are in contrast not only with our findings, but also with the original hypothesis of the authors. Notably, if we carefully compare this with our analysis, several differences can be evidenced, providing an explanation for this discrepancy. First, there is a different period of analysis since O’Shea et al. looked to a 100-day period starting from 21 January until 30 April, while we adopted the official lockdown period according to Italian public restriction measures (9 March to 3 May) (see Figure 5). The variability in restriction measures in the U.S. report during the study period may limit the association of arrhythmic burdens with lockdown measures. Moreover, the first control period (i.e., 12 September to 20 December 2019) of the U.S. cohort included autumn and winter, which are more associated with arrhythmias according to available reports [22]. More importantly, as correctly acknowledged by O’Shea et al., the U.S. report provides data limited to the incidence of ventricular arrhythmias without other clinical or remote monitoring data, limiting the possibility to correlate their findings with limitation of physical activity. Finally, patients in the first period after a CIED implant were not excluded, thus affecting the incidence of ventricular arrhythmias, especially in responders to cardiac resynchronization therapy [21]. Finally, the possibility to include in our analysis the evaluation of the parameters among the same cohort of patients during the post-lockdown period reinforces our results compared to previous studies. Taken together, our findings and the results of O’Shea et al. clearly evidence that a pandemic can have a great impact on the arrhythmic profiles of CIED patients through different mechanisms beyond infection [23]. Available data should be carefully analyzed to provide recommendations on patient management during future lockdown measures to mitigate possible negative cardiovascular effects.

## 5. Limitations

The major limitation of our clinical research is that, deriving from a project aiming at observations of device use in standard clinical practice, it has an observational non-randomized design. Besides this, the limitations of multicentre observational studies, such as potential biases in patient selection or patient treatment, apply to our research. However, we believe that the acknowledged limitations were mitigated by the prospective data collection, the exclusion of patients with recent CIED implantation, and the availability of a control period for both the lockdown and post-lockdown periods. Moreover, the uniformity of restrictive measures that applied to the whole country, coupled with a great difference in the spread of the COVID-19 virus among different regions strengthen our findings. Our analysis may only provide a hypothesis on the effect of restrictive measures on patients implanted with ICD/CRT devices since a detailed collection of visits and drug treatments has not been included in the analyzed database.

## 6. Conclusions

In a large Italian cohort of CIED carriers, we found an increase in the burden of atrial fibrillation and ventricular arrhythmias coupled with a decrease in physical activity and heart rate variability during the lockdown period for the COVID-19 pandemic. The findings were similar in areas with low and high prevalence of COVID-19 infection, suggesting a negative impact of the COVID-19 pandemic beyond direct infection, probably related to restrictions connected with containment of the pandemic. Future studies are warranted to confirm our findings, while providing suggestions to plan remote support for cardiovascular rehabilitation and psychological distress.

## Figures and Tables

**Table 1:** Characteristics of the enrolled population.

Clinical Characteristics	(N = 696)
**Age at first implant (yrs),** Mean ± SD	63.7 ± 13.2
**Time from first implant to 4 March 2019 (yrs),** Mean ± SD	4.85 ± 3.62
**Gender (Male),** %, n/Pts	78.0% (536/687)
**Therapy,** %, n/Pts	
**Dual chamber**	17.8% (124/696)
**Biventricular**	59.3% (413/696)
**Single chamber**	21.0% (146/696)
**Other/Unknown**	1.9% (13/696)
**Prevention,** %, n/Pts	
**Primary**	68.1% (474/696)
**Secondary**	15.1% (105/696)
**Other/Unknown**	16.8% (117/696)
**Medical history,** %, n/Pts	
**History of HF**	70.0% (471/673)
**NYHA Class 3/4**	37.9% (232/612)
**History of VT/VF**	32.6% (220/675)
**History of AT/AF**	29.1% (197/677)
**Paroxysmal AF**	17.4% (118/677)
**Persistent AF**	3.7% (25/677)
**Permanent AF**	8.0% (54/677)
**MI**	31.4% (214/681)
**Third grade AV block**	6.2% (43/696)
**LBBB**	38.2% (266/696)
**Sinus Node Disease**	7.6% (49/646)
**History of syncope**	14.2% (78/549)
**History of Stroke/TIA**	6.0% (35/586)
**Hypertension**	58.1% (377/649)
**Diabetes**	23.8% (145/609)
**Chronic Kidney Disease**	11.7% (76/652)
**COPD**	11.0% (65/591)
**CHADS2 ≥ 2,** %, n/Pts	58.8% (306/520)
**CHADS2-VASC ≥ 4,** %, n/Pts	31.5% (98/311)
**LVEF at baseline (%),** Mean ± SD	33.7 ± 12.1

Legend: AT/AF = atrial tachycardia/atrial fibrillation; AV = atrio-ventricular; COPD = chronic obstructive pulmonary disease; HF = heart failure; LVEF = left ventricular ejection fraction; LBBB = left bundle branch block; MI = myocardial infarction; NYHA = New York Heart Association; Pts = patients; SD = standard deviation; TIA = transient ischemic attack; VT/VF = ventricular tachycardia/ventricular fibrillation. CHADS2 and CHADS2-VASC are two standard scores adopted for stratification of thromboembolic risk of atrial fibrillation.

**Table 2:** Baseline clinical characteristics and follow-up parameters in patients with vs. without AT/AF and/or VT/VF during lockdown period.

Clinical Characteristics	AT/AF < 1 h and no VT/VF Arrythmias (N = 595)	AT/AF ≥ 1 h a/o VT/VF Arrythmias (N = 101)	*p*-Value
**Age at first implant (yrs), Mean ± SD**	63.2 ± 13.4	66.5 ± 11.2	0.044
**Time from first implant to 4 March 2019 (yrs),** Mean ± SD	4.90 ± 3.65	4.57 ± 3.45	0.321
**Gender (Male),** %, n/Pts	77.9% (457/587)	79.0% (79/100)	0.798
**Therapy,** %, n/Pts			
**Dual chamber**	17.6% (105/595)	18.8% (19/101)	0.242
**Biventricular**	58.5% (348/595)	64.4% (65/101)	
**Single chamber**	22.2% (132/595)	13.9% (14/101)	
**Other/Unknown**	1.7% (10/595)	3.0% (3/101)	
**Prevention,** %, n/Pts			
**Primary**	69.1% (411/595)	62.4% (63/101)	0.309
**Secondary**	15.0% (89/595)	15.8% (16/101)	
**Other/Unknown**	16.0% (95/595)	21.8% (22/101)	
**Medical history,** %, n/Pts			
**History of HF**	69.5% (398/573)	73.0% (73/100)	0.476
**NYHA Class 3/4**	38.1% (200/525)	36.8% (32/87)	0.815
**History of VT/VF**	31.0% (179/577)	41.8% (41/98)	0.035
**History of AT/AF**	24.3% (140/577)	57.0% (57/100)	<0.001
**Paroxysmal AF**	13.9% (80/577)	38.0% (38/100)	<0.001
**Persistent AF**	2.1% (12/577)	13.0% (13/100)	<0.001
**Permanent AF**	8.3% (48/577)	6.0% (6/100)	0.429
**MI**	30.3% (177/585)	38.5% (37/96)	0.105
**3rd grade AV block**	6.2% (37/595)	5.9% (6/101)	0.915
**LBBB**	38.2% (227/595)	38.6% (39/101)	0.930
**Sinus Node Disease**	7.1% (39/552)	10.6% (10/94)	0.226
**History of syncope**	13.6% (63/463)	17.4% (15/86)	0.350
**History of Stroke/TIA**	5.4% (27/500)	9.3% (8/86)	0.158
**Hypertension**	57.0% (314/551)	64.3% (63/98)	0.177
**Diabetes**	23.2% (121/521)	27.3% (24/88)	0.410
**Chronic Kidney Disease**	11.4% (63/554)	13.3% (13/98)	0.590
**COPD**	57.0% (252/442)	69.2% (54/78)	0.043
**CHADS2 ≥ 2, %,** n/Pts	31.7% (83/262)	30.6% (15/49)	0.883
**CHADS2-VASC ≥ 4,** %, n/Pts	10.5% (53/505)	14.0% (12/86)	0.343
**LVEF at baseline (%),** Mean ± SD	34.4 ± 13.1	31.4 ± 7.9	0.706
**Follow-up**			
**Average Median activity** (min), Mean ± SD	152.0 ± 111.9	123.8 ± 109.5	0.008
**Average Median heart rate variability** (ms), Mean ± SD	87.0 ± 31.6	82.6 ± 44.4	0.136
**Average Number of days with Optivol threshold > 60**	52.8 ± 143.5	87.7 ± 194.1	0.004
**Average Number of days with Optivol threshold > 120**	25.3 ± 109.6	50.6 ± 165.2	<0.001

Legend: AT/AF = atrial tachycardia/atrial fibrillation; AV = atrio-ventricular; COPD = chronic obstructive pulmonary disease; HF = heart failure; LVEF = left ventricular ejection fraction; MI = myocardial infarction; NYHA = New York Heart Association; Pts = patients; LBBB = left bundle branch block; SD = standard deviation; TIA = transient ischemic attack; VT/VF = ventricular tachycardia/ventricular fibrillation. CHADS2 and CHADS2-VASC are two standard scores adopted for stratification of thromboembolic risk of atrial fibrillation.

## Data Availability

Underlying data will be made available by the corresponding authors upon reasonable request.

## References

[1] Cheng W., Zhang Z., Cheng W., Yang C., Diao L., Liu W. (2018). Associations of leisure-time physical activity with cardiovascular mortality: A systematic review and meta-analysis of 44 prospective cohort studies. Eur. J. Prev. Cardiol..

[2] Kivimäki M., Singh-Manoux A., Pentti J., Sabia S., Nyberg S.T., Alfredsson L., Goldberg M., Knutsson A., Koskenvuo M., Koskinen A. (2019). Physical inactivity, cardiometabolic disease, and risk of dementia: An individual-participant meta-analysis. BMJ.

[3] Lippi G., Henry B.M., Sanchis-Gomar F. (2020). Physical inactivity and cardiovascular disease at the time of Coronavirus disease 2019 (COVID-19). Eur. J. Prev. Cardiol..

[4] Dherange P., Lang J., Qian P., Oberfeld B., Sauer W.H., Koplan B., Tedrow U. (2020). Arrhythmias and COVID-19. JACC Clin. Electrophysiol..

[5] Impatto Dell’Epidemia COVID-19 Sulla Mortalità Totale della Popolazione Residente Periodo Gennaio-Novembre 2020. https://www.iss.it/documents/20126/0/Rapp_Istat_Iss_FINALE+2020_rev.pdf/b4c40cbb-9506-c3f6-5b69-0ccb5f015172?t=1609328171264.

[6] Biffi M., Candelora A., Massaro G. (2017). Ventricular fibrillation triggered by earthquake during the accumoli-amatrice disaster in Italy. Circ. J..

[7] Nakano M., Kondo M., Wakayama Y., Kawana A., Hasebe Y., Shafee M.A., Fukuda K., Shimokawa H. (2012). Increased incidence of tachyarrhythmias and heart failure hospitalization in patients with implanted cardiac devices after the great East Japan earthquake disaster. Circ. J..

[8] Soumya R.S., Unni T.G., Raghu K.G. (2021). Impact of COVID-19 on the cardiovascular system: A review of available reports. Cardiovasc. Drugs.

[9] Official Declaration of the COVID-19 Pandemic by the WHO the 11th March 2020. https://www.who.int/director-general/speeches/detail/who-director-general-s-opening-remarks-at-the-media-briefing-on-covid-19---11-march-2020.

[10] Gupta A.K., Jneid H., Addison D., Ardehali H., Boehme A.K., Borgaonkar S., Boulestreau R., Clerkin K., Delarche N., DeVon H.A. (2020). Current perspectives on Coronavirus disease 2019 and cardiovascular disease: A white paper by the JAHA Editors. JAHA.

[11] Kulkarni P., Mahadevappa M., Alluri S. (2020). COVID-19 pandemic and the impact on the cardiovascular disease patient care. CCR.

[12] Bromage D.I., Cannatà A., Rind I.A., Gregorio C., Piper S., Shah A.M., McDonagh T.A. (2020). The impact of COVID-19 on heart failure hospitalization and management: Report from a heart failure unit in London during the peak of the pandemic. Eur. J. Heart Fail..

[13] Boukhris M., Hillani A., Moroni F., Annabi M.S., Addad F., Ribeiro M.H., Mansour S., Zhao X., Ybarra L.F., Abbate A. (2020). Cardiovascular implications of the COVID-19 pandemic: A global perspective. Can. J. Cardiol..

[14] Rattka M., Baumhardt M., Dreyhaupt J., Rothenbacher D., Thiessen K., Markovic S., Rottbauer W., Imhof A. (2020). 31 Days of COVID-19—Cardiac events during restriction of public life—A comparative study. Clin. Res. Cardiol..

[15] Boriani G., Palmisano P., Guerra F., Bertini M., Zanotto G., Lavalle C., Notarstefano P., Accogli M., Bisignani G., AIAC Ricerca Network Investigators (2020). Impact of COVID-19 pandemic on the clinical activities related to arrhythmias and electrophysiology in Italy: Results of a survey promoted by AIAC (Italian Association of Arrhythmology and Cardiac Pacing). Intern. Emerg. Med..

[16] Lakkireddy D.R., Chung M.K., Gopinathannair R., Patton K.K., Gluckman T.J., Turagam M., Cheung J., Patel P., Sotomonte J., Lampert R. (2020). Guidance for cardiac electrophysiology during the COVID-19 pandemic from the Heart Rhythm Society COVID-19 Task Force; Electrophysiology section of the american college of cardiology; and the Electrocardiography and Arrhythmias Committee of the Council on Clinical Cardiology, American Heart Association. Circulation.

[17] Tarantini G., Fraccaro C., Chieffo A., Marchese A., Tarantino F.F., Rigattieri S., Limbruno U., Mauro C., La Manna A., Castiglioni B. (2020). Italian Society of Interventional Cardiology (GISE) position paper for cath lab-specific preparedness recommendations for healthcare providers in case of suspected, probable or confirmed cases of COVID-19. Catheter. Cardiovasc. Interv..

[18] Mahmud E., Dauerman H.L., Welt F.G.P., Messenger J.C., Rao S.V., Grines C., Mattu A., Kirtane A.J., Jauhar R., Meraj P. (2020). Management of acute myocardial infarction during the COVID-19 pandemic: A consensus statement from the Society for Cardiovascular Angiography and Interventions (SCAI), the American College of Cardiology (ACC), and the American College of Emergency Physicians (ACEP). Catheter. Cardiovasc. Interv..

[19] Van der Velde E.T., Atsma D.E., Foeken H., Witteman T.A., Hoekstra W.H. (2013). Remote monitoring of patients with implanted devices: Data exchange and integration. Eur. J. Prev. Cardiol..

[20] Boriani G., De Ponti R., Guerra F., Palmisano P., Zanotto G., D’Onofrio A., Ricci R.P. (2021). Sinergy between drugs and devices in the fight against sudden cardiac death and heart failure. Eur. J. Prev. Cardiol..

[21] O’Shea C.J., Thomas G., Middeldorp M.E., Harper C., Elliott A.D., Ray N., Lau D.H., Campbell K., Sanders P. (2021). Ventricular arrhythmia burden during the Coronavirus disease 2019 (COVID-19) pandemic. Eur. Heart J..

[22] Russo V., Solimene F., Zanotto G., Pisanò E.C., Della Bella P., Iacopino S., Pignalberi C., Calvi V., Maglia G., Quartieri F. (2019). Seasonal trend of ventricular arrhythmias in a nationwide remote monitoring database of implantable defibrillators and cardiac resynchronization devices. Int. J. Cardiol..

[23] Boriani G., Vitolo M. (2021). COVID-19 pandemic: Complex interactions with the arrhythmic profile and the clinical course of patients with cardiovascular disease. Eur. Heart J..

